# Patterns of genetic variation among geographic and host-plant associated populations of the peach fruit moth *Carposina sasakii* (Lepidoptera: Carposinidae)

**DOI:** 10.1186/s12862-017-1116-7

**Published:** 2017-12-20

**Authors:** You-Zhu Wang, Bing-Yan Li, Ary Anthony Hoffmann, Li-Jun Cao, Ya-Jun Gong, Wei Song, Jia-Ying Zhu, Shu-Jun Wei

**Affiliations:** 1 Institute of Plant and Environmental Protection, Beijing Academy of Agriculture and Forestry Sciences, 9 Shuguanghuayuan Middle Road, Haidian District, Beijing, 100097 China; 2 Key Laboratory of Forest Disaster Warning and Control of Yunnan Province, College of Forestry, Southwest Forestry University, Kunming, 650224 China; 3 School of BioSciences, Bio21 Institute, The University of Melbourne, Melbourne, VIC 3010 Australia

**Keywords:** *Carposina sasakii*, Microsatellite, Mitochondrial gene, Host-associated differentiation, Population genetic structure

## Abstract

**Background:**

Populations of herbivorous insects may become genetically differentiated because of local adaptation to different hosts and climates as well as historical processes, and further genetic divergence may occur following the development of reproductive isolation among populations. Here we investigate the population genetic structure of the orchard pest peach fruit moth (PFM) *Carposina sasakii* (Lepidoptera: Carposinidae) in China, which shows distinct biological differences when characterized from different host plants. Genetic diversity and genetic structure were assessed among populations from seven plant hosts and nine regions using 19 microsatellite loci and a mitochondrial sequence.

**Results:**

Strong genetic differentiation was found among geographical populations representing distinct geographical regions, but not in host-associated populations collected from the same area. Mantel tests based on microsatellite loci indicated an association between genetic differentiation and geographical distance, and to a lesser extent environmental differentiation. Approximate Bayesian Computation analyses supported the scenario that PFM likely originated from a southern area and dispersed northwards before the last glacial maximum during the Quaternary.

**Conclusions:**

Our analyses suggested a strong impact of geographical barriers and historical events rather than host plants on the genetic structure of the PFM; however, uncharacterized environmental factors and host plants may also play a role. Studies on adaptive shifts in this moth should take into account geographical and historical factors.

**Electronic supplementary material:**

The online version of this article (10.1186/s12862-017-1116-7) contains supplementary material, which is available to authorized users.

## Background

Genetic variation among natural populations can develop due to a number of factors that include geographical isolation, ecological isolation and historical processes. Geographical barriers that limit dispersal and consequently lead to isolation by distance (IBD) appear to be particularly important in population divergence in diverse taxa [1]. However, extant patterns of genetic differentiation may also be impacted by historical processes, such as those associated with climate oscillations of the Quaternary, when many species became restricted to refugia in glacial periods, interspersed by range expansions in interglacial periods [2]. And in the last decade, an increasing number of studies have shown that ecological factors also play an important role in shaping genetic differentiation (isolation by environment, IBE) [3–7].

The relative contribution of these factors on population differentiation can be difficult to determine [3]. In empirical and simulation studies, false positives or underestimated correlations between genetic and environmental variations can be generated through the influence of IBD and spatial autocorrelation of ecological variables [5]. In Mantel tests of IBD, hierarchical population structure, which is mostly caused by postglacial recolonisation from multiple refugia, can be confounded with IBE [8]. A better understanding of the complex factors influencing population differentiation needs well designed sampling srategies, and a combined consideration of geography, history and ecology [9].

Herbivorous insects represent a diverse group of species with a wide range of distributions and adaptive potential [10–13]. Population genetic differentiation of these insects may be influenced by geographical, historical and ecological factors [9, 14]. Host plants represent one obvious form of ecological variation that can play a crucial role in the diversification of herbivorous insect populations [15]. Alternative host-plant species can generate different selection pressures that create ecological barriers to gene flow between insect populations [16–18]. Because hosts often differ in traits that are linked ecologically and physiologically to performance (e.g. nutritional quality, recognition cues), fitness trade-offs and divergent selection between plants can occur and contribute to ecological isolation and speciation [19–21]. An increasing number of cases of host-associated differentiation have been documented in insects [11, 22–27].

The peach fruit moth (PFM), *Carposina sasakii* Matsumura (Lepidoptera: Carposinidae), is a major phytophagous orchard pest widely distributed in Northeast Asia [28]. Larvae of PFM bore into the fruits of multiple hosts in the Rosaceae and Rhamnaceae, mainly apple, pear, peach, apricot, hawthorn, Chinese quince, jujube, and wild jujube. On different host plants, PFMs vary in performance both under field and laboratory conditions. In the field, peak adult emergence time, oviposition habitat and generation number can vary (Table S1), likely to synchronize developmental stages with hosts. Under laboratory conditions, adult PFMs live significantly longer on jujube than on other hosts, adult females reared from jujube and peach tend to lay more eggs [29], and larval survival also varies with host plant [30]. Phenological isolation associated with host usage may facilitate host-associated adaptation and reduce flow among host-associated populations. Based on biological observations, esterase isozyme patterns [31] and random amplified polymorphic DNA (RAPD) [32], PFMs on different hosts have been proposed as representing host biotypes. Based on mtDNA, two sympatric and cryptic lineages of the PFM were identified in populations from China; however, no association between population variation and host plants was found [33].

Geographical isolation may also contribute to genetic differentiation of PFM. Based on variation in the mtDNA *cox1* gene, there is a correlation between genetic differentiation and geographical distance [33]. Using 35 microsatellite loci, genetic differentiation was detected between two geographically distant populations collected from two host plants, Chinese quince and apple [34]. Both host usage and geographic isolation might therefore contribute to genetic differentiation in PFM.

In this study, we simultaneously characterized genetic variation of PFM from both host-associated and geographical populations across China, using microsatellite markers and mtDNA. We hypothesized that populations from different host plants would differ genetically when the influence of geographical influence was removed, and also that populations from different geographical locations would show IBD given the wide distribution range of this species. We therefore estimated the degree of genetic differentiation of PFM associated with different hosts versus geographic distance and also considered historical factors. Our study sheds light on understanding ecological and evolutionary processes that drive divergence of PFM and the possibility of host-associated reproductive isolation in this species.

## Methods

### Specimen collection and DNA extraction

**Fig. 1:**
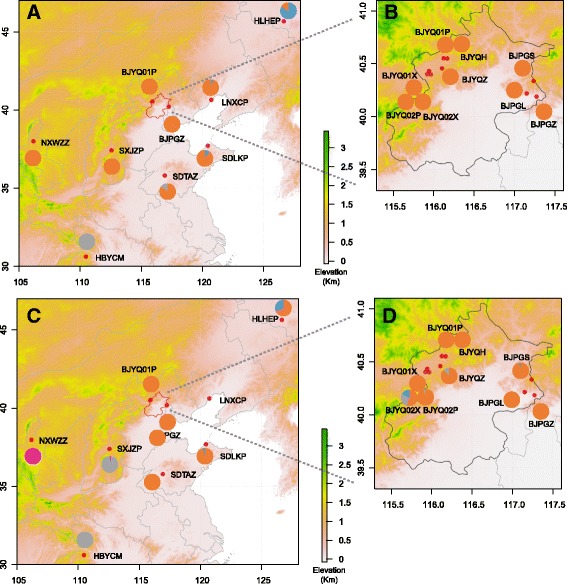
Collection sites of *Carposina sasakii* and BAPS analysis of geographical and host-associated populations based on microsatellite loci and mtDNA. The different colors in each population correspond to the frequency of cluster membership based on the BAPS analysis. Figs **a** and **c** show separation in the geographical populations, where one cluster and two separate populations were identified based on microsatellite loci (Fig. **a**), while based on mtDNA four clusters were identified (Fig. **c**). Figs **b** and **d** show the host-associated populations, which provided no strong evidence of genetic structure based either on microsatellites (Fig. **b**) or mtDNA (Fig. **d**)

In total 410 PFM larvae were sampled from damaged fruits of host plants in 16 populations with permissions from the orchard owners (Table 1 and Fig. 1). The 10 host-associated populations were collected from seven hosts of apple, pear, hawthorn, apricot, crabapple, Chinese quince (Rosaceae) and jujube (Rhamnaceae). The nine geographical populations cover most of the distribution of PFM in China. To separate geographical distance from host plant effects, nine host-associated populations were collected from Beijing in northern China, as well as a population from Chinese quince collected from Hubei province in southern China, 865 km from Beijing. We included two populations from jujube, apple and apricot in Beijing to evaluate genetic differentiation between populations from the same host plant. The distance among host-associated populations in Beijing ranged from adjacent orchards (BJYQ02X and BJYQ02P) to a distance of 151 km. Samples were obtained from multiple trees at each location, stored in absolute ethanol and frozen at −80 °C prior to DNA extraction. Genomic DNA was extracted from a segment of individual larva using DNeasy Blood & Tissue Kit (QIAGEN, Hilden, Germany).

**Table 1:** Sample collection information for the *Carposina sasakii* used in this study

Group	Population	Collection location	Longitude (°E)	Latitude (°N)	Collection date	Host plant	No.
H1	BJPGL	Pinggu, Beijing	117.1504	40.2159	30/09/2011	Pear (*Pyrus* spp.)	24
H2	BJPGS	Pinggu, Beijing	117.2369	40.3337	29/09/2011	Hawthorn (*Crataegus* spp.)	24
H3, G1	BJPGZ	Pinggu, Beijing	117.2731	40.1859	19/09/2012	Jujube (*Ziziphus jujuba*)	24
H4	BJYQH	Yanqing, Beijing	116.1697	40.5452	15/09/2012	Crabapple (*Malus* spp.)	24
H5	BJYQZ	Yanqing, Beijing	116.1011	40.4737	10/09/2012	Jujube (*Ziziphus jujuba*)	24
H6, G2	BJYQ01P	Yanqing, Beijing	115.9458	40.5247	16/09/2012	Apple (*Malus pumila*)	31
H7	BJYQ01X	Yanqing, Beijing	115.9164	40.4319	08/07/2016	Apricot (*Armeniaca vulgaris*)	15
H8	BJYQ02P	Yanqing, Beijing	115.9164	40.4319	/07/2017	Apple (*Malus pumila*)	23
H9	BJYQ02X	Yanqing, Beijing	115.9164	40.4319	/07/2017	Apricot (*Armeniaca vulgaris*)	24
H10, G3	HBYCM	Yichang, Hubei province	110.5108	30.6171	17/6/2012	Chinese quince (*Chaenomeles speciosa*)	32
G4	HLHEP	Haerbin, Heilongjiang province	126.6663	45.6417	01/09/2011	Apple (*Malus pumila*)	32
G5	LNXCP	Huludao, Liaoning province	120.7442	40.6193	01/10/2012	Apple (*Malus pumila*)	32
G6	NXWZZ	Wuzhong, Ningxia province	106.2195	37.9811	19/09/2016	Jujube (*Ziziphus jujuba*)	12
G7	SDLKP	Yantai, Shandong province	120.4747	37.7021	01/10/2012	Apple (*Malus pumila*)	29
G8	SDTAZ	Taian, Shandong province	116.9467	35.7899	01/08/2013	Jujube (*Ziziphus jujuba*)	32
G9	SXJZP	Jinzhong, Shanxi province	112.5972	37.3952	17/10/2013	Apple (*Malus pumila*)	28

H1-H10, eight host-associated populations; G1-G9, nine geographical populations; No., number of individuals used in the study

### Microsatellite genotyping and mtDNA sequencing

For the nuclear markers, we genotyped 19 polymorphic microsatellite loci from each individual, developed with methods used in our previous study [34] (Table S2). This involved using PC tail (Primer tail C) modified forward primers and fluorescence-labeled PC tails (FAM, HEX, and ROX) for amplification [35]. For the mitochondrial marker, a fragment of mitochondrial *cox1* gene (507 bp) was amplified using primer pair LCO1490 and HCO2198 [36]. Polymerase chain reaction (PCR) was conducted using the Mastercycler pro system (Eppendorf, Germany) with standard PCR conditions and an annealing temperature of 52 °C. Amplified products were purified and sequenced directly from both strands using an ABI 3730xl DNA Analyzer (Applied Biosystems, USA).

### Genetic diversity analyses

Prior to population genetic analysis, microsatellites genotyped by GENEMAPPER version 4.0 (Applied Biosystems, USA) were checked for stuttering, scoring error, large allele dropout and presence of null alleles by MICRO-CHECKER [37]. Allele frequencies, number of alleles, observed (*H*_*O*_) and expected (*H*_*E*_) heterozygosity, were estimated by macros in Microsatellite Tools [38]. Null allele frequency was estimated using FREENA [39] with 10,000 bootstraps. In addition, deviations from Hardy-Weinberg Equilibrium (HWE) and tests for linkage disequilibrium (LD) were calculated with an exact probability test [40] implemented in GENEPOP version 4.0 [41].

Sequencing results of mtDNA from both strands were assembled. Amino acid sequences were aligned by codons using CLUSTALW [42] implemented in MEGA version 6 [43] under default parameters. Nucleotide sequence alignment was guided by aligned amino acid sequences. The number of polymorphic sites (*S*), total number of mutations (η), number of haplotypes (*H*), haplotype diversity (*Hd*), nucleotide diversity (*Pi*), nucleotide diversity with Jukes and Cantor correction *Pi* (JC), Tajima’s D and average number of nucleotide differences (*K*) were calculated with DnaSP version 5.0 [44].

### Population structure analysis

For microsatellites loci, genetic differentiation among 14 populations of PFM was measured by pairwise F_*ST*_ calculated in FREENA version 4.0 with ENA [41]. For mtDNA, ARLEQUIN suit version 3.5 was used to conduct an exact test of population differentiation based on default parameters [45].

In order to incorporate spatial information into clustering of individuals, the BAPS (Bayesian analysis of population structure) model implemented in software BAPS version 6.0 [46] was used based on microsatellite loci or mtDNA. For microsatellite data, the number of populations (*K*) ranged from 1 to 20 with 20 iterations per *K* value, while for mtDNA, 20 runs (*K* = 20, 15 and 10) were performed to ensure convergence and consistency of the results.

We performed a Discriminant Analysis of Principal Components (DAPC) analysis using adegenet 1.4–2 implemented in R [47] based on microsatellite loci, which plots individuals in space based on genetic similarity without biological assumption.

### Isolation by distance and environment

In order to evaluate the effect of geographic distance on genetic differentiation of host-associated populations in Beijing region and assess the level of isolation by distance (IBD) within geographical populations, a Mantel test correlating genetic distance (F_*ST*_/(1-F_*ST*_)) and geographic distance was undertaken using ade4 version 1.7–4 implemented in R (Daniel et al. 2004) with 999 replicates. The values of F_*ST*_ were calculated in FREENA version 4.0 with ENA [41] for microsatellite data and ARLEQUIN suit version 3.5 for mtDNA (Excoffier & Lischer 2010).

To check the influence of environmental factors on population genetic differentiation, the presence of isolation by environment (IBE) was tested. Firstly, 19 bioclimatic variables were downloaded from WorldClim database (http://www.worldclim.org/) using the getData function implemented in R package RASTER. Subsequently, we extracted corresponding bioclimatic values of each location using the getData function. Three vegetation variables (NDVI: normalized difference vegetation index, LAI: leaf area index, and percent tree cover) were downloaded from MODIS landcover database (https://modis-land.gsfc.nasa.gov/) and then extracted with ArcGIS multiple version 10.2 (ESRI Inc., Redlands, CA). Then we extracted bioclimatic values and vegetation values of each location using cbind function in R package and ARCGISmultiple. Finally, a principal component analysis was performed to analyze the 22 environmental variables for each locality using prcomp function in R. The first two principal components were used to estimate environmental distances between locations. Environmental distances were compared with genetic distances (F_*ST*_/(1-F_*ST*_)) based on microsatellite or mtDNA by Mantel tests in R package ade4 version 1.7–4 with 999 replicates.

To investigate the extent of eco-spatial autocorrelation in our data, we performed a Mantel test between the ecological and geographical distance matrices. To further assess the relative contribution of environmental variables and geographical distance, matrix regression with a randomization (MMRR) method implemented in R with 10,000 permutations was used [48].

### Haplotype relationship analysis and molecular dating

Haplotype relationships were constructed through the software SPLITSTREE version 4.13.1 [49], while the divergence times for haplotype lineages were estimated using the software BEAST version 1.8.1 [50], as described in [51]. In molecular dating analysis, *Carposina fernadana* and *Carposina hyperlopha* were used as outgroups.

### Test on scenarios of PFM dispersal

**Fig. 2:**
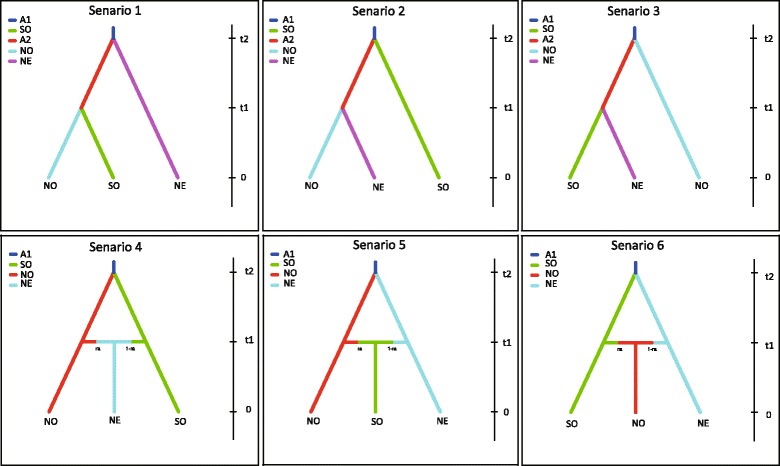
Graphical representation of the six scenarios for the three population groups. NE, northeast populations; NO, north populations; SO, south populations. A1 and A2 are two unknown (ghost) populations divided into two branches. Scenario 1, 2 and 3 correspond to possible evolutionary relationships among the three populations without admixture between any two of them. Scenario 4, 5 and 6 assume that one of the three populations is an admixture of two other populations

The approximate Bayesian computation (ABC) method implemented in DIYABC version 2.1.0 [52] was followed to compare different dispersal scenarios and infer the ancestral populations in PFM based on microsatellite loci (Fig. 2). Datasets were generated by selecting different populations representing the identified groups of PFM, in order to avoid misleading results and false signals of bottlenecks caused by pooling different samples to identify a group, and simplifying complexity of scenarios to be compared [53, 54]. In total, two datasets were provided in the analysis. Moreover, we assumed two unknown populations as ghost populations divided into two branches. In total, six biologically plausible dispersal scenarios representing the relationships of the three groups were conducted and compared, considering the variation of population size and the split and admixture events. The six scenarios could be split into two categories with or without admixture events. Details of pre-evaluation scenario-prior combinations, estimation of posterior distributions of parameters, model checking, and evaluations of confidence in scenario choice are described in supporting information (Additional file 1: Appendix S1).

## Results

### Genetic diversity and pairwise population differentiation

All microsatellite loci used in the study proved to be polymorphic. The mean number of alleles for each population was high, and the *H*_*O*_ was similar to *H*_*E*_ in each population. The host-associated and geographically separated populations showed similar values for genetic diversity parameters (Table S3). There was no obvious LD among the 19 microsatellite loci; no loci were significantly linked or departed from HWE across all populations, and no population departed from HWE across all loci.

In total 35 haplotypes were observed (GenBank accession numbers: KY492475-KY492509), among which 14 haplotypes were uniquely represented by one individual, 10 shared among individuals but not across populations, and 11 shared among different populations. Tajima’s D was not significantly different from 0 in all populations (Table S4) after Holm’s correction [55].

Null alleles did not generate bias in estimates of population differentiation (Additional file 1: Appendix S2). Fewer populations were genetically differentiated between pairs of host-associated populations collected in Beijing than between pairs of geographically isolated populations (Table 2). F_*ST*_ values between the geographically separated Chinese quince population and other populations were mostly higher than those between the other population (Table 2).

**Table 2:** Pairwise F_*ST*_ values of comparisons among 14 *Carposina sasakii* populations based on microsatellite loci (lower triangle) and mtDNA (upper triangle)

Population	BJPGL	BJPGS	BJPGZ	BJYQH	BJYQZ	BJYQ01P	BJYQ01X	BJYQ02P	BJYQ02X	HBYCM	HLHEP	LNXCP	NXWZZ	SDLKP	SDTAZ	SXJZP
BJPGL		−0.0151	−0.0214	−0.0097	0.0146	0.0015	0.0157	−0.0311	0.2094**	0.7508**	0.1881	0.0019	0.8389**	0.2001*	0.0473	0.8347**
BJPGS	0.0056		−0.0065	0.0016	0.0295	0.0107	−0.0048	−0.0094	0.2159**	0.7294**	0.1902	0.0053	0.8018**	0.2158**	0.0607	0.8120**
BJPGZ	0.0100	0.0090		−0.0097	0.049	0.0018	0.0656	−0.0277	0.2335**	0.7719**	0.2081	−0.0096	0.8679**	0.2263*	0.0812	0.8538**
BJYQH	0.0079	0.0023	0.0026		0.043	−0.0235	0.0436	−0.0215	0.2458**	0.7912**	0.2088	0.0059	0.9013**	0.2180*	0.036	0.8739**
BJYQZ	0.0139	0.0065	0.0249**	0.0056		0.0138**	0.0526**	−0.0026	0.1486**	0.7019**	0.0841	0.0674	0.7576**	0.1419	0.0436	0.7875**
BJYQ01P	0.0152**	0.0166**	0.0040	0.0051	0.0462		0.0405	−0.0139	0.2733**	0.7979**	0.2155*	0.0025	0.8942**	0.2040*	0.0092	0.8738**
BJYQ01X	0.0287**	0.0325**	0.0257*	0.0186*	0.036	0.0235**		0.023	0.2448**	0.8027**	0.2138	0.049	0.9723**	0.2800*	0.1018	0.9006**
BJYQ02P	0.0394**	0.0265**	0.0227**	0.0065**	0.0209**	0.0206**	0.0252**		0.1905**	0.7475**	0.1541*	−0.004	0.8314**	0.1728**	0.0301	0.8316**
BJYQ02X	0.0571**	0.0437**	0.0359**	0.0209**	0.0266**	0.0307**	0.0497**	−0.0005		0.5298**	0.0828*	0.2883**	0.4534**	0.1658**	0.2643**	0.6052**
HBYCM	0.0857**	0.0773**	0.0934**	0.0722**	0.0776**	0.0837**	0.0997**	0.0903**	0.0916**		0.5648**	0.7894**	0.7101**	0.7150**	0.8016**	0.3771**
HLHEP	0.0575**	0.0629**	0.0647**	0.0399**	0.0730**	0.0792**	0.0631**	0.0604**	0.0760**	0.1073**		0.2357*	0.5407**	0.11	0.1974	0.6508**
LNXCP	0.0201**	0.0108**	0.0123**	0.0072**	0.0274**	0.0252**	0.0204**	0.0300**	0.0451**	0.0908**	0.0384**		0.8767**	0.2463**	0.0731	0.8634**
NXWZZ	0.0443**	0.0521**	0.0551**	0.0497**	0.0614**	0.0542**	0.0479**	0.0464**	0.0717**	0.1055**	0.0828**	0.0538**		0.7641**	0.8970**	0.8409**
SDLKP	0.0249**	0.0111**	0.0357**	0.0161**	0.0272**	0.0331**	0.0370**	0.0487**	0.0607**	0.0933**	0.0587**	0.0105**	0.0713**		0.1426	0.7888**
SDTAZ	0.0282**	0.0223**	0.0383**	0.0269**	0.0256**	0.0378**	0.0560**	0.0481**	0.0596**	0.0533**	0.0624**	0.0271**	0.0663**	0.0151**		0.8763**
SXJZP	0.0490**	0.0378**	0.0284**	0.0146**	0.0236**	0.0267**	0.0307**	0.0006**	0.0160**	0.0875**	0.0606**	0.0327**	0.0442**	0.0481**	0.0490*	

* indicates *P* < 0.05, ** indicates *P* < 0.01 following Holm’s correction

### Population genetic structure

For the nine geographical populations, BAPS analysis based on microsatellite loci revealed that seven populations clustered into one large group, while one northern population and one southern population were separated from this cluster with minor admixture (Fig. 1a). The analysis based on mtDNA identified four groups, which did not entirely coincide with the microsatellite groups (Fig. 1c). Most individuals in the nine Beijing populations collected from different hosts fell into one major cluster for both types of markers (Fig. 1b and d).

**Fig. 3:**
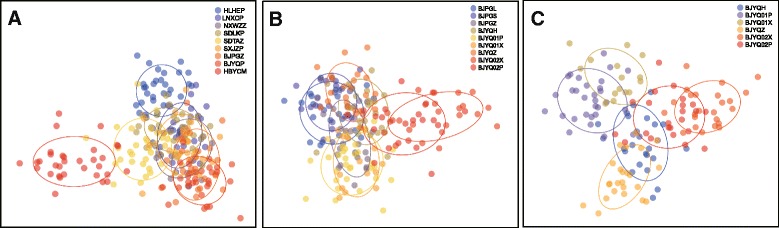
Discriminant Analysis of Principal Components (DAPC) in populations of *Carposina sasakii*. **a** Nine geographic populations from different regions in China (**b**) Nine host-associated populations collected from Beijing. **c** Six host-associated populations collected from Yanqing of Beijing

DAPC analyses indicated genetic differentiation between the southern population collected on Chinese quince and other populations within the nine geographical populations (Fig. 3a). No differentiation was found among nine host-associated populations collected from two areas of Beijing (Fig. 3b) or six host-associated populations collected from the Yanqing area of Beijing (Fig. 3c).

### Isolation by geographical and environmental distances

Both microsatellite and mitochondrial data showed a lack of any association between host-associated population differentiation and distance in the Beijing region (*r* = −0.056, *P* = 0.677 for microsatellite loci, *r* = −0.014, *P* = 0.502 for mtDNA). For the microsatellite data, Mantel tests indicated the presence of both IBD and IBE when considering the geographically separated populations of PFM. However, a significant correlation between ecological and geographical distance was found (*r* = 0.767, *P* = 0.002). The standardized regression coefficient for geographic distance onto genetic distance based on all populations (*β*_*D*_ = 0.476, *P* = 0.0080) was similar to the equivalent regression coefficient for environmental distance (*β*_*D*_ = 0.443, *P* = 0.0115), suggesting that IBD was stronger than IBE. For the mtDNA, there was no evidence of either IBD or IBE.

### Haplotype network, divergence time and demographic history

**Fig. 4:**
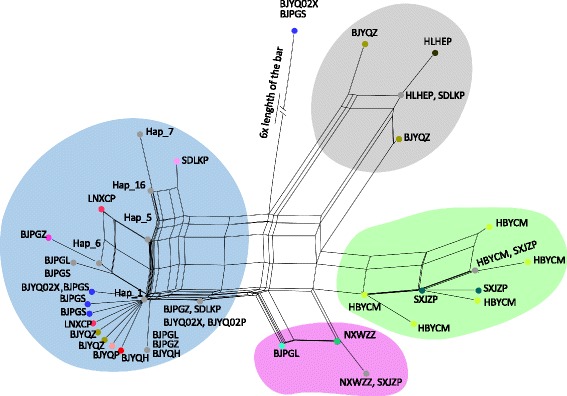
The SPLITSTREE network from 14 *Carposina sasakii* collections based on mtDNA. Four major lineages were found. The largest one included haplotypes mainly from northeastern populations (blue). The second lineage was composed of haplotypes from southern China (green). The remaining two lineages are mainly composed of haplotypes from the northeast population (grey) and the western population (pink), with minor contributions from the other populations. Points in the same color (except for grey) indicate haplotypes from the same population. Points in grey indicate haplotypes shared by populations. The points labeled by hap_1, hap_5, hap_6, hap_7 and hap_16 were haplotypes shared by individuals from northern populations

SPLITSTREE analysis divided the mitochondrial haplotypes into four major lineages (Fig. 4), mostly corresponding to the four geographical groups identified in the population genetic structure analyses but with some admixture. The southern and western lineages were more closely related to each other than to the other lineages. One haplotype from an eastern population (BJPGL) fell into the western lineage.

Molecular clock analysis of the mtDNA indicated that an ancient haplotype diverged from others 1.02 Ma (million years ago) with a 95% highest posterior density (HPD) of 0.43–1.83 (Additional file 1: Figure S1). Two major lineages, corresponding to the southern and western lineages versus northern and northeastern lineages, diverged 0.70 Ma (95% HPD = 0.35–1.15), while the divergence times within the two lineages were 0.52 (95% HPD = 0.24–0.87) and 0.39 (95% HPD = 0.15–0.70) Ma, respectively (Additional file 1: Figure S1).

### Dispersal routes

The ABC analyses supported scenario 2 (posterior probability of 0.3768 on average) as the most likely based on microsatellite data (Fig. 2). In this scenario, the southern population and A2 (a ghost population) are from A1 (another ghost population), and A2 is a population established later and linked to the northeastern and northern populations. The choice of scenarios was reliable based on an evaluation of confidence and model checking (Additional file 1: Appendix S1).

## Discussion

### Geographical but no host-associated differentiation

Our analyses revealed strong genetic differentiation among geographical populations of PFM, but no host-associated differentiation. The genetic clusters correspond to different geographical regions, indicating a strong effect of geographical barriers on population divergence in PFM. Although some clusters were identified by only a single population, the population genetic structure analysis is congruent with the phylogenetic network pattern. Geographical isolation plays an important role in population divergence in nature [1, 4] and geographically structured populations have been documented in two other orchard insects which also cause heavy damage to fruit [14, 56]. Geographically differentiated pest populations might be more likely to occur in pests of orchards compared to those in ephemeral crop and vegetable fields [51, 57, 58] due to the relatively stable ecosystem provided by orchards [59].

There was a clear lack of host-associated differentiation in PFM based on collections from nine populations in the Beijing area. These populations included two collected from apple with a genetic distance higher than some distances obtained from population pairs from different hosts (Table 2). The lowest F_*ST*_ value (−0.0005) came from the pair of populations collected from adjacent apple and apricot orchards (BJYQ02P and BJYQ02X, Table 2). The Chinese quince population showed strong genetic differentiation between other host-associated populations. However, this population was geographically separate from the other populations. Two other populations from jujube (NXWZZ and SDTAZ) and one from apple (SXJZP) were also geographically separated (Fig. 1c), and the population from Chinese quince grouped with a population from apple based on mtDNA. Each of the two populations from jujube, apple and apricot separated into two different clusters, further highlighting the lack of plant host effect on genetic differentiation. However, we could not exclude the occasions of locally formed host-associated differentiation out of the study area.

Previous studies have investigated host-associated differentiation of PFM [31–33, 60]. Based on esterase isozymes of three host populations, Hua and Hua argued for differentiation between populations collected from apple and populations from jujube and wild jujube; however, the distance between collection locations was about 500 km [31]. RAPD marker data also suggested genetic differentiation linked to hosts and particularly apricot in comparison with apple, hawthorn, peach, cornel, jujube, and wild jujube [32]. This pattern contrasts sharply with the lack of differentiation found here, which might reflect the markers used [61] or the nature of the populations tested. Perhaps esterases detect adaptive differences associated with selection. Nevertheless, the very low genetic differentiation between hosts in adjacent orchards suggests high gene flow between hosts, at least in northern China where this sampling took place. Sympatric host-associated populations from other geographical areas are needed to validate the absence of host-associated differentiation in PFM.

A high level of gene flow in PFM populations may prevent host-related differentiation even if there is host-associated selection. PFM adults tend to be highly mobile [62] and therefore move between adjacent orchards. It is also possible that there is some genetic differentiation among host types, but this was not detected because the markers we used are not involved in host plant adaptation. Biological studies have suggested host differences in the induction of diapause and temperature-dependent development in PFM [62] and these may reflect differentiation at loci under selection. Genes related to biological characteristics, such as circadian clock genes, metabolic arrest, adult eclosion, host selection and oviposition behavior may be differentiated [63]. These might not result in genetic differentiation at neutral markers, particularly if adaptive differentiation is very recent [64, 65]. New technologies targeting genome-wide differentiation may be needed for detecting host-associated genetic differentiation of PFM involving adaptive loci [66].

### Historical events and population differentiation

Molecular dating revealed an early divergence of the mtDNA about 1 Ma (within Pleistocene, 2.58–0.0117 Ma), pointing to an influence of climatic vacillation during the Quaternary on PFM. This suggests that PFM may be useful for testing hypotheses about the historical effects of the Quaternary on phylogeographical patterns in China, with patterns found so far contrasting with those of well-studied areas of Europe and North America [2, 67]. During the glacial periods of Quaternary, no unified ice sheet had developed in China [68], providing opportunities for divergence and even regional expansion of organisms before the last glacial maximum (LGM, 0.018–0.025 Ma) [14, 69]. Few studies on insects have traced these patterns of divergence [14], unlike plant studies that have shown evidence for multiple refugees in China (east Asia) during the Quaternary [67], mostly located in the southern region [70], but also in the northern region [71].

We explored all possible hypotheses on the origin and dispersal of PFM based on the identified genetic groups using the ABC method. This method is suitable to test complex scenarios in population genetics [72], and has been used in recent work [14, 53, 73, 74]. Our analyses support the notion that the PFM originated from southern China followed by dispersal from south to north. In terms of pest management, PFM was considered as a major pest of deciduous fruit trees in northern China, although damage was occasionally found in southern China. Southern China was warmer than northern regions during the Quaternary, likely allowing species to persist there. A similar pattern of origin and dispersal has been reported in another orchard pest, *Grapholita molesta* [14].

Molecular dating showed the divergence time of major lineages before 0.39 Ma, indicating colonization of northern China by PFM before LGM. This is congruent with the pattern for *G. molesta* in China [14].

### Isolation by distance and environment

Apart from IBD, habitats can contribute to genetic divergence by creating barriers to gene flow [75], resulting in IBE [3]. Mantel tests based on microsatellite data showed the presence of both IBD and IBE in populations of PFM. High false positive rates for Mantel tests of IBE can arise when high levels of IBD and eco-spatial autocorrelation occurs [76], and Mantel tests showed strong correlations in our data. However, while the MMRR analysis suggested that the effect of geographical isolation on genetic differentiation was stronger than environmental factors, there was also an effect of IBE in PFM populations, in support of patterns in the literature that suggest IBE is common [4, 5]. In PFM, the emergence of adults, development rate and voltinism depend on temperature [77, 78], and the sampled populations cover a wide geographical range along a temperature gradient. With temperature affecting life history traits, relatively higher rates of gene flow might be expected across populations sharing a similar thermal environment. This might be tested further by comparing patterns of gene flow across topographically complex areas where a high degree of local temperature variation might be present.

It is unclear why there was an apparent hierarchical structure of mtDNA variation in PFM, which did not appear connected to IBD or IBE. Incongruent population structure between mitochondrial and nuclear genes has been noted in many studies [79, 80], and could be due to an incomplete natural history of the mitochondrial genome due to a range of factors such as a small effective population size, high mutation rate and patterns of introgression [81]. High differentiation based on mitochondrial genes but low based on microsatellite loci indicated complicated population history, such as the existence of multiple refugia populations during glacial periods followed by admixture in the interglacial periods, as reported in other species [14, 54]. Admixture of clusters was noted in several populations identified by BAPS analysis, suggesting ongoing introgression or incomplete lineage sorting. This was further supported by the SPLITSTREE analysis on mtDNA, in which haplotypes from the southern population (HBYCM) and one of its nearby population (SXJZZ) clustered in the same lineage.

## Conclusions

Based on microsatellite loci and mtDNA, we found strong genetic differentiation in populations of PFM, but no obvious evidence for host-associated differentiation in PFM involving its common plant hosts, even though these plants alter PFM phenology and life history. Our study suggests that the geographical isolation and historical events in the Quaternary had a strong impact on current genetic differentiation of PFM in China. These strong effects may conceal other factors such as host-associated adaption and the impact of local environmental conditions. While host-associated adaptation of PFM might be present, it is not sufficient to generate separate gene pools of PFM that might reflect incipient speciation. Our study also suggests that geographical and historical factors need to be considered in experimental designs when attempting to assess adaptive divergence in PFM. Taking advantage of genomic tools, there are opportunities to investigate these processes further by incorporating a high density of markers across the genome that might include markers linked to loci under selection [82, 83].

## Additional file

Additional file 1: Appendix S1. Analysis of dispersal scenarios of *Carposina sasakii* using approximate Bayesian computation (DIYABC). **Appendix S2.** Power analysis of the microsatellite markers used in the study and effect of null alleles on population differentiation estimation. **Figure S1.** Phylogenetic trees of the haplotypes of the peach fruit moth *Carposina sasakii* based on mtDNA. The population name(s) followed by the haplotype indicates the haplotype found in the population(s). The value near the node indicates the corresponding divergence time (million years ago) of the two branches. In addition, the values of divergence times in red are given with a 95% highest posterior density (HPD). **Table S1.** Summary information on the biology of *Carposina sasakii* on different host plants. **Table S2.** Microsatellite loci used in this study. **Table S3.** Summary statistics for diversity of the 19 microsatellite loci examined in 14 populations of *Carposina sasakii.* **Table S4.** Genetic diversity of *Carposina sasakii* populations based on mitochondrial *cox1* gene. (DOCX 741 kb)

## Abbreviations

ABC: approximate Bayesian computation
BAPS: Bayesian analysis of population structure
*cox1*: cytochrome c oxidase subunit I
DAPC: discriminant analysis of principal components
ENA: excluding null alleles
HEW: Hardy-Weinberg Equilibrium
HKY: Hasegawa-Kishino-Yano
IBD: isolation by distance
IBE: isolation by environment
K2P: Kimura-2-parameter
LD: linkage disequilibrium
LGM: last glacial maximum
MMRR: multiple matrix regression with randomization
mtDNA: mitochondrial DNA
PFM: peach fruit moth
