# Supplementary material for: Patterns of genetic variation among geographic and host-plant associated populations of the peach fruit moth Carposina sasakii (Lepidoptera: Carposinidae)
Source: BMC Evol Biol. 2017 Dec 20;17:265. doi: 10.1186/s12862-017-1116-7 (PMC5738824; doi:10.1186/s12862-017-1116-7)
Supplement: Additional file 1: Appendix S1. — Analysis of dispersal scenarios of Carposina sasakii using approximate Bayesian computation (DIYABC). Appendix S2. Power analysis of the microsatellite markers used in the study and effect of null alleles on population differentiation estimation. Figure S1. Phylogenetic trees of the haplotypes of the peach fruit moth Carposina sasakii based on mtDNA. The population name(s) followed by the haplotype indicates the haplotype found in the population(s). The value near the node indicates the corresponding divergence time (million years ago) of the two branches. In addition, the values of divergence times in red are given with a 95% highest posterior density (HPD). Table S1. Summary information on the biology of Carposina sasakii on different host plants. Table S2. Microsatellite loci used in this study. Table S3. Summary statistics for diversity of the 19 microsatellite loci examined in 14 populations of Carposina sasakii. Table S4. Genetic diversity of Carposina sasakii populations based on mitochondrial cox1 gene. (DOCX 741 kb) [file 12862_2017_1116_MOESM1_ESM.docx]

**Additional Files**

**Patterns of** **genetic variation among geographic and host-plant associated populations of the peach fruit moth *Carposina* *sasakii* (Lepidoptera: Carposinidae)**

**Appendix S1** Analysis of dispersal scenarios of *Carposina sasakii* using approximate Bayesian computation (DIYABC)

**Appendix S2** Power analysis of the microsatellite markers used in the study and effect of null alleles on population differentiation estimation

**Fig. S1** Phylogenetic trees of the haplotypes of the peach fruit moth *Carposina sasakii* based on mtDNA. The population name(s) followed by the haplotype indicates the haplotype found in the population(s). The value near the node indicates the corresponding divergence time (million years ago) of the two branches. In addition, the values of divergence times in red are given with a 95% highest posterior density (HPD).

**Table S1** Summary information on the biology of *Carposina sasakii* on different host plants

**Table S2** Microsatellite loci used in this study

**Table S3** Summary statistics for diversity of the 19 microsatellite loci examined in 16 populations of *Carposina sasakii*

**Table S4** Genetic diversity of *Carposina sasakii* populations based on mitochondrial *cox1* gene

**Appendix S1** Analysis of dispersal scenarios of *Carposina sasakii* using approximate Bayesian computation (DIYABC)

**Method:**

Scenarios for dispersal routes of *C. sasakii* were tested by using DIYABC based on microsatellite loci. First, six competing scenarios among the three population groups (SO, NO and NE) identified based on microsatellite loci were tested (Fig. 2 in main text). Second, four competing scenarios derived from the best scenario of the last step were compared (Fig. III). Datasets were generated by selecting a single population (BJPGZ or BJYQP) to represent the largest cluster (NO) identified by BAPS, in order to avoid misleading results and false signals of bottlenecks caused by pooling different samples to identify a group. This simplifies complexity in the scenarios to be compared and balances data used for each cluster ([Cao *et al.* 2016](#_ENREF_2); [Lombaert *et al.* 2014](#_ENREF_10)).

Prior parameters were set as in Table I. In total 10^6^ simulations were performed for all 36 summary statistics including 12 one-sample summary statistics and 24 two-sample statistics. Only 1% of simulated datasets closest to the observed data were used to compare the competing scenarios based on corresponding posterior probabilities using logistic regression with a linear discriminant analysis ([Estoup *et al.* 2012](#_ENREF_6)). Posterior distributions of parameters selected above used the closest 1% simulated datasets ([Cornuet *et al.* 2008](#_ENREF_5)). Confidence in the scenario choice was evaluated by a logistic approach. Model checking of the selected scenario was performed to evaluate goodness-of-fit with 1% of the simulated datasets closest to our real data based on all summary statistics ([Cornuet *et al.* 2010](#_ENREF_4)).

**Results:**

In the first step, scenario 2 was considered the best (posterior probability is 0.3768 on average) with high confidence (Type I/II error was 0.47/0.28 on average). The South group and A2 (a ghost population) are from A1 (another ghost population), and A2 is established later consisting of the northeast and north groups (Fig. 2 in main text). In the second step, the two datasets generated inconsistent results. When the populations of HBYCM, BJPGZ and HLHEP were used, scenario 2 was the optimal one (Fig. II-B, posterior probability is 0.3909), but when HBYCM, BJYQP and HLHEP were used, scenario 3 (Fig. III-C, posterior probability is 0.3083) was the optimal one. Therefore, our results suggest a south group of *C. sasakii* split off earlier when compared to the northern and northeast groups.

**Table I** Prior distributions for DIYABC analysis of *Carposina sasakii* and the posterior parameter estimations for scenario 2 in step 1. There were no obvious differences in the prior distribution results between the datasets. Posterior distributions were generated based on populations of HBYCM, BJPGZ and HLHEP.

| Parameter | Prior distributions | Posterior distribution | |
| --- | --- | --- | --- |
|  |  | Median | 95% CI |
| A1 | 10-10000 | 1.84E+03 | 5.78E+02-4.45E+03 |
| A2 | 10-20000 | 6.08E+03 | 1.11E+03-1.74E+04 |
| HL | 10-6000 | 3.65E+03 | 1.41E+03-5.62E+03 |
| PG | 10-5000 | 1.34E+03 | 3.42E+02-3.87E+03 |
| HB | 10-5000 | 2.74E+03 | 1.21E+03-4.44E+03 |
| t1 | 10-500 | 1.92E+02 | 4.86E+01-4.10E+02 |
| t2 | 10-1000 | 5.84E+02 | 2.52E+02-9.31E+02 |
| µmic_1 | 1.00E-004-1.00E-3 | 1.91E-04 | 1.13E-04-4.41E-04 |
| pmic_1 | 1.00E-001-3.00E-001 | 2.63E-01 | 1.84E-01-3.00E-01 |
| snimic_1 | 1.00E-009-1.00E-005 | 1.07E-07 | 1.16E-08-2.72E-06 |

A1 and A2 represent two ghost populations; t1 and t2 are the first observed outbreak time; µmic_1, pmic_1 and snimic_1 represent mutations model of microsatellite data.


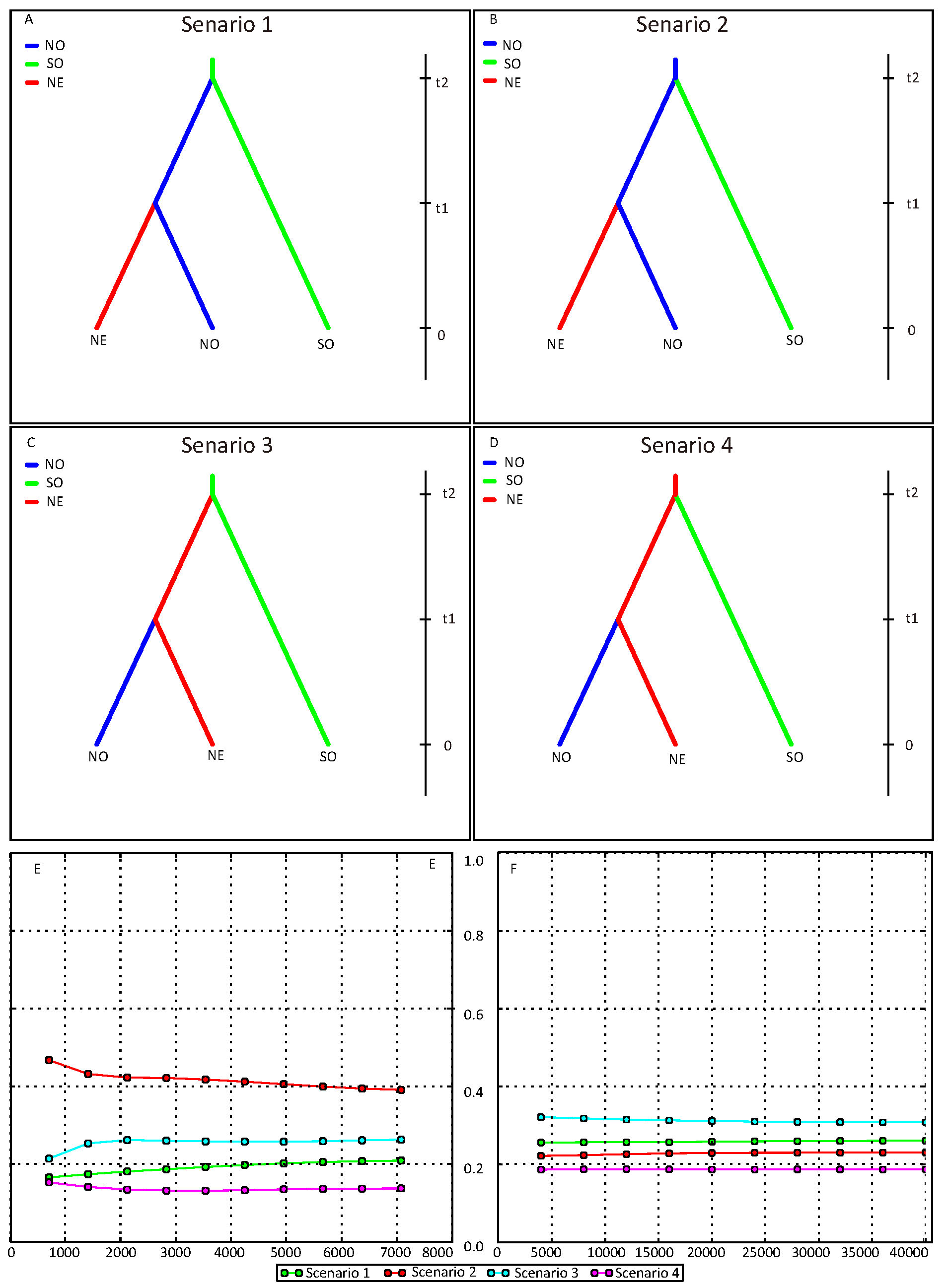


**Fig. III** Scenarios for *Carposina sasakii* dispersal routes (A-D) and logistic regression plots showing the posterior probability of each scenario for dataset (E) (HBYCM, BJPGZ and HLHEP) and (F) (HBYCM, BJYQH and HLHEP) from DIYABC analysis based on microsatellite loci.

**Appendix S2** Power analysis of the microsatellite markers used in the study and effect of null alleles on population differentiation estimation

**Methods:**

In order to assess the statistical power affected by optional combinations including the number of samples, sample sizes, number of loci and alleles as well as allele frequencies, POWSIM version 4.1 ([Ryman & Palm 2006](#_ENREF_11)) was used, which is a simulation based computer program that estimates power using chi-square and Fisher’s exact tests when evaluating the hypothesis of genetic homogeneity and degree of differentiation. A total of 2000 replicates were performed using population sizes and allele frequencies from the data, an effective population size of 4000 and drift generations varying from 8 to 80 to obtain expected F*_ST_* values from 0.001 to 0.01.

Exact tests for population differentiation were performed by FREENA ([Chapuis & Estoup 2007](#_ENREF_3)). FREENA generated two sets of F*_ST_* value, one excluding null alleles (the ENA method), the other including the effect of null alleles ([Chapuis & Estoup 2007](#_ENREF_3)). To check the effect of null alleles on estimation of differentiation, the two sets of F*_ST_* values were compared by a repeatability analysis, treating each comparison as a sample and then running an ANOVA. This test is appropriate for directional bias since it does not test whether the two approaches give similar F*_ST_* values, only if one is consistently larger or smaller.

**Results:**

The POWSIM analysis showed that the 19 microsatellite loci are sufficient to provide a 99.95% probability of detecting an F*_ST_* as low as 0.005 for all populations, 96.60% probability for nine host associated populations from Beijing, and 86.30% probability for six host-associated populations from Yanqing in Beijing. All of the 91 F*_ST_* values calculated from the 14 populations were higher than 0.005 except for three comparisons among host-associated populations, indicating that the microsatellite markers and sample sizes used in our study could have detected the level of differentiation we observed among the sampled populations.

The repeatability value was 0.99, meaning that about 99% of the variation between F*_ST_* or F*_ST_* corrected by ENA were due to differences among different pairwise population comparisons. Therefore, no significant difference between F*_ST_* and F*_ST_* corrected by ENA was detected, indicating that null alleles did not generate bias in estimates of differentiation.


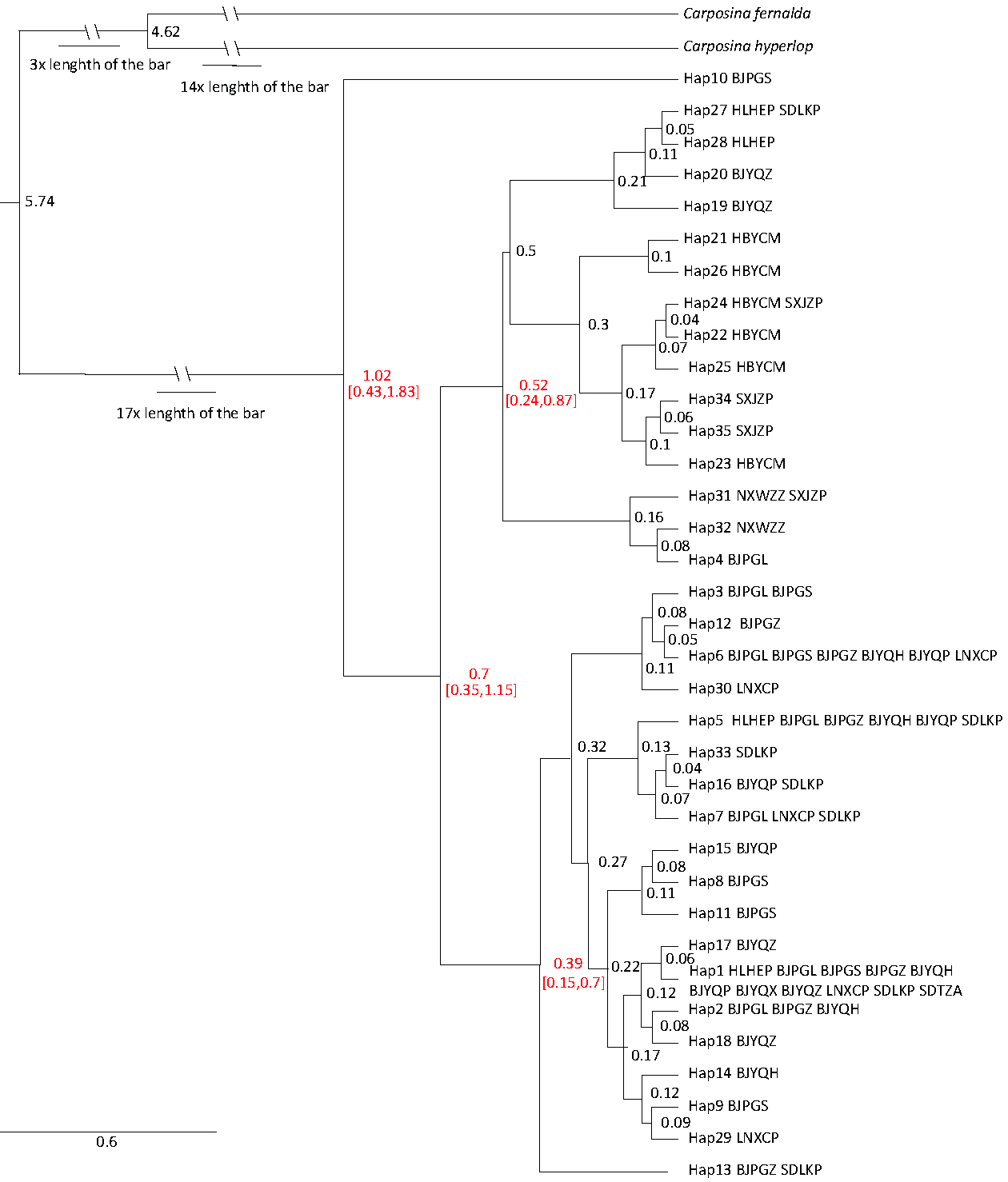


**Fig. S1** Phylogenetic trees of the haplotypes of the peach fruit moth *Carposina sasakii* based on mtDNA. The population name(s) followed by the haplotype indicates the haplotype found in the population(s). The value near the node indicates the corresponding divergence time (million years ago) of the two branches. In addition, the values of divergence times in red are given with a 95% highest posterior density (HPD).

**Table S1** Summary information on the biology of *Carposina sasakii* on different host plants

| Host plant | Fruit development period | Adult peak of emergence time | Oviposition habitat | Generations | Reference |
| --- | --- | --- | --- | --- | --- |
| Apple | Mid-Jun. to Sept. | Mid-Jun., Mid-Aug., (Early Sept) | Fruit depression | 1-2 | ([Hua & Hua 1995](#_ENREF_8)) |
| Jujube/wild jujube | Mid-Jul. to mid-Sept. | Mid-Jul., Late Aug. | Leaf back | 1-2 | ([Hua & Hua 1995](#_ENREF_8)) |
| Pear | Late Jun. to Sept. | Early Aug. | Fruit depression | 1 | ([Fang 2016](#_ENREF_7); [Hua & Hua 1995](#_ENREF_8)) |
| Pomegranate | Early Jun. to Oct. | Early Jul., Early Aug., Late Aug. | Fruit surface | 3 | ([Cai & Ding 1990](#_ENREF_1); [Hua & Hua 1995](#_ENREF_8)) |
| Apricot | Late Apr. to Jun. | Early Jun. to Mid-Jun. | Fruit calyx | 1 | ([Hua & Hua 1995](#_ENREF_8); [Hua *et al.* 1998](#_ENREF_9)) |

**Table S2** Microsatellite loci used in this study developed by [Wang *et al.* (2016)](#_ENREF_12)

| Locus | Dye | Repeat motif | Forward primer sequence (5’-3’) | Reverse primer sequence (5’-3’) | Tm/°C |
| --- | --- | --- | --- | --- | --- |
| CS03 | ROX | (AGT)6 | TAAAAGCGATTCGTTGGGAC | ATGGCGTCATATCTTCGACC | 56 |
| CS04 | FAM | (ACT)6 | TTCCGTGCATGTCGTAAGAG | CGCGTTTAGCATCAATCTCA | 56 |
| CS06 | HEX | (CCG)6 | ACCGACCAGTCCATTCGAT | CTCCTTAGGTCTCTGCGTCG | 56 |
| CS07 | HEX | (AAT)6 | AGCAGCCTGCATCCAACC | ACACACTCCCAATTCGCTTC | 56 |
| CS101 | HEX | (AAC)6 | TTGGTTCATGGATCTAGGAGG | TCCTAAGTCTACCTAACTTTATGTGTT | 56 |
| CS102 | HEX | (AGT)6 | CCGTAATAATTCGACACAAGCA | CCTATACTCGTATACTTAAACAACTGA | 56 |
| CS103 | FAM | (AAC)6 | AGTATCAAAAGAAACCCCTAA | ATCGGCATTATTTGTAAGGT | 56 |
| CS11 | HEX | (AAG)6 | CCTCGTATTAGATTAGGCGGAA | CCCAAGTTGAATGGGAACAG | 56 |
| CS14 | HEX | (AGT)6 | TGCGACAAAATGCCAGAATA | GCCGATGTATTCTAATGAAGCC | 56 |
| CS19 | HEX | (AGT)6 | CCAATGTGTCGTACAACGTG | CCTCAAGTAAATATAATCAGGGCG | 56 |
| CS20 | HEX | (ACT)6 | CAAATCCTTGGCAATGTGAA | AGAAAAGATTCACCTGCGCT | 56 |
| CS22 | HEX | (CCG)6 | GTAACGAGCGCAATTGATGA | CGCGCTAATCTGGTTAATACG | 56 |
| CS28 | HEX | (ACT)6 | GCTGGTGTGGATGGCATAGT | AACTTCGAATTTCCATTGCG | 56 |
| CS33 | FAM | (ACT)6 | AATAGGGCTCCTCCACACCT | GATCTGCAAATCTGCCTGTG | 56 |
| CS34 | FAM | (AGT)6 | CGCCCTAGACGAACCTACAC | GCCTATGTTCAGCAGAAGACG | 56 |
| CS35 | FAM | (AAG)6 | CAAAGATAATGTACAAAGACGTG | CAACTGTCTGCAACACAGCA | 56 |
| CS36 | ROX | (CCG)6 | CACCGATTTGTTTTATCGCA | GGCGCTAATGTCTACCCTCA | 56 |
| CS41 | FAM | (AAC)6 | CCACTGGGCTATCACTGCTAT | TGCAACAGTGACATCACAAGA | 56 |
| CS47 | FAM | (AGT)7 | ACCGGTATTGCTGTATTTGT | CAATTTGTGATTAGGTATTTGTTTCAA | 56 |

| **Table S3** Summary statistics for diversity of the 19 microsatellite loci examined in 14 populations of *Carposina sasakii*Group | Population | Sample size | Mean number of alleles | Observed heterozygosity | Expected heterozygosity |
| --- | --- | --- | --- | --- | --- |
| H1 | BJPGL | 24 | 3.95 | 0.4132 | 0.4943 |
| H2 | BJPGS | 24 | 4.00 | 0.4502 | 0.5013 |
| H3, G1 | BJPGZ | 24 | 3.63 | 0.4439 | 0.4890 |
| H4 | BJYQH | 24 | 4.42 | 0.4144 | 0.5072 |
| H5 | BJYQZ | 24 | 4.26 | 0.4248 | 0.5047 |
| H6, G2 | BJYQ01P | 31 | 4.47 | 0.3957 | 0.4961 |
| H7 | BJYQ01X | 15 | 3.37 | 0.4169 | 0.4787 |
| H8 | BJYQ02P | 23 | 3.74 | 0.4205 | 0.4856 |
| H9 | BJYQ02X | 24 | 3.42 | 0.3839 | 0.4677 |
| H10, G3 | HBYCM | 32 | 4.16 | 0.3579 | 0.4565 |
| G4 | HLHEP | 32 | 4.79 | 0.3992 | 0.5070 |
| G5 | LNXCP | 32 | 4.05 | 0.3809 | 0.4769 |
| G6 | NXWZZ | 12 | 3.05 | 0.3604 | 0.4815 |
| G7 | SDLKP | 29 | 4.21 | 0.3614 | 0.4605 |
| G8 | SDTAZ | 32 | 3.95 | 0.4191 | 0.4732 |
| G9 | SXJZP | 28 | 3.89 | 0.3442 | 0.4585 |

H1-H10, eight host-associated populations; G1-G9, nine geographical populations; No., number of individuals used in the study.

**Table S4** Genetic diversity of *Carposina sasakii* populations based on mitochondrial *cox1* gene

| Group | Population | *S* | η | *H* | *Hd* | *Pi* | *Pi*(JC） | *K* | *D* |
| --- | --- | --- | --- | --- | --- | --- | --- | --- | --- |
| H1 | BJPGL | 9 | 9 | 7 | 0.605 | 0.00220 | 0.00221 | 1.116 | -1.76000 |
| H2 | BJPGS | 15 | 15 | 7 | 0.558 | 0.00290 | 0.00293 | 1.471 | -2.23953 |
| H3, G1 | BJPGZ | 5 | 5 | 6 | 0.681 | 0.00177 | 0.00178 | 0.899 | -0.95316 |
| H4 | BJYQH | 4 | 4 | 5 | 0.540 | 0.00121 | 0.00122 | 0.616 | -1.16225 |
| H5 | BJYQZ | 10 | 10 | 5 | 0.377 | 0.00337 | 0.00340 | 1.710 | -1.20571 |
| H6, G2 | BJYQ01P | 4 | 4 | 5 | 0.488 | 0.00128 | 0.00128 | 0.649 | -0.89428 |
| H7 | BJY01QX | 0 | 0 | 1 | 0.000 | 0.00000 | 0.00000 | 0.000 | 0.00000 |
| H8 | BJYQ02P | 9 | 9 | 6 | 0.537 | 0.00239 | 0.00240 | 1.212 | -1.70619 |
| H9 | BJYQ02X | 14 | 14 | 4 | 0.699 | 0.01047 | 0.01059 | 5.308 | 1.45673 |
| H10, G3 | HBYCM | 6 | 6 | 6 | 0.0647 | 0.00301 | 0.00302 | 1.524 | 0.06475 |
| G4 | HLHEP | 8 | 8 | 4 | 0.504 | 0.00629 | 0.00635 | 3.190 | 1.81272 |
| G5 | LNXCP | 5 | 5 | 5 | 0.514 | 0.00156 | 0.00156 | 0.790 | -6.97542 |
| G6 | NXWZZ | 1 | 1 | 2 | 0.303 | 0.00060 | 0.00060 | 0.303 | -0.19492 |
| G7 | SDLKP | 9 | 9 | 7 | 0.778 | 0.00368 | 0.00370 | 1.867 | -0.58042 |
| G8 | SDTAZ | 2 | 2 | 2 | 0.315 | 0.00124 | 0.00124 | 0.629 | 0.53579 |
| G9 | SXJZP | 6 | 6 | 4 | 0.563 | 0.00171 | 0.00171 | 0.865 | -1.27445 |

Note: *S*, number of polymorphic (segregating) sites; η, total number of mutations; *H*, number of haplotypes; *Hd*, haplotype diversity; *Pi*, nucleotide diversity; *K*, average number of nucleotide differences; *Pi*(JC), nucleotide diversity with Jukes and Cantor correction; *D*, Tajima's D; All values of Tajima's D were not significant (*P* > 0.05) following Holm’s correction.

**References**

Cai P, Ding WZ (1990) Briefing of peach fruit moth damage on pomegranate. *China Fruits*, 35-37.

Cao LJ, Wei SJ, Hoffmann AA, Wen JB, Chen M, Vaclavik T (2016) Rapid genetic structuring of populations of the invasive fall webworm in relation to spatial expansion and control campaigns. *Diversity and Distributions*, **22**, 1276-1287.

Chapuis MP, Estoup A (2007) Microsatellite null alleles and estimation of population differentiation. *Molecular Biology & Evolution*, **24**, 621-631.

Cornuet JM, Ravigné V, Estoup A (2010) Inference on population history and model checking using DNA sequence and microsatellite data with the software DIYABC (v1.0). In: *BMC Bioinformatics*, p. 11.

Cornuet JM, Santos F, Beaumont MA, Robert C, Marin J-M, Balding DJ, Guillemaud T, Estoup A (2008) Inferring population history with DIY ABC: a user-friendly approach to Approximate Bayesian Computation. *Bioinformatics*, **24**, 2713-2829.

Earl DA, vonHoldt BM (2011) STRUCTURE HARVESTER: a website and program for visualizing STRUCTURE output and implementing the Evanno method. *Conservation Genetics Resources*, **4**, 359-361.

Estoup A, Lombaert E, Marin JM, Guillemaud T, Pudlo P, Robert CP, Cornuet JM (2012) Estimation of demo-genetic model probabilities with Approximate Bayesian Computation using linear discriminant analysis on summary statistics. *Molecular Ecology Resources*, **12**, 846-855.

Fang CG (2016) The occurrence regularity and pollution-free control technology of peach fruit moth *Caposina sasakii* (Lepidoptera: Carposinidae) in Xinbin mountains. *Protection Forest Science and Technology*, 109-110.

Hua L, Hua BZ (1995) Preliminary study on the host-biotypes of peach fruit borer. *Acta Phytophylacica Sinica*, **22**, 165-170.

Hua L, Hua BZ, huang WL (1998) The bionomics of peach fruit borer damaged on apricot tree. *Acta Phytophylacica Sinica*, **25**, 141-144.

Jakobsson M, Rosenberg NA (2007) CLUMPP: a cluster matching and permutation program for dealing with label switching and multimodality in analysis of population structure. *Bioinformatics*, **23**, 1801-1806.

Jombart T, Devillard S, Dufour AB, Pontier D (2008) Revealing cryptic spatial patterns in genetic variability by a new multivariate method. *Heredity*, **101**, 92-103.

Lombaert E, Guillemaud T, Lundgren J, Koch R, Facon B, Grez A, Loomans A, Malausa T, Nedved O, Rhule E, Staverlokk A, Steenberg T, Estoup A (2014) Complementarity of statistical treatments to reconstruct worldwide routes of invasion: the case of the Asian ladybird *Harmonia axyridis*. *Molecular Ecology*, **23**, 5979-5997.

Pritchard JK, Stephens M, Donnelly P (2000) Inference of population structure using multilocus genotype data. *Genetics*, **7**, 574–578.

Rosenberg NA (2003) Distruct: a program for the graphical display of population structure. *Molecular Ecology Notes*, **4**, 137-138.

Ryman N, Palm S (2006) POWSIM: a computer program for assessing statistical power when testing for genetic differentiation. *Molecular Ecology Notes*, **6**, 600-602.

Wang YZ, Cao LJ, Zhu JY, Wei SJ (2016) Development and characterization of novel microsatellite markers for the peach fruit moth *Carposina sasakii* (Lepidoptera: Carposinidae) using next-generation sequencing. *International Journal of Molecular Sciences*, **17**, 362.

Cai P, Ding WZ (1990) Briefing of peach fruit moth damage on pomegranate. *China Fruits*, 35-37.

**Abstract**: The peach fruit moth, *Carposina sasakii* Matsumura (named *Carposina niponensis* wals. in the past) (Lepidoptera: Carposinidae), is a pest on apple, pear, jujube and peach fruit. But there is no report on damage on pomegranate (*Punica granatum* L.) In recent years, serious harm of peach fruit moth to pomegranate in Huaiyuan County of Anhui province and other areas. For example, in Huaiyuan County, since 1985, fruit decay rate on pomegranate has reached about 80%. In order to clear the occurrence of the pest on pomegranate in Anhui province and find out better management, we carried out observation and controlling experiments in 1986 and 1987 in two places Huaiyuan and Shouxian.

Cao LJ, Wei SJ, Hoffmann AA, Wen JB, Chen M, Vaclavik T (2016) Rapid genetic structuring of populations of the invasive fall webworm in relation to spatial expansion and control campaigns. *Diversity and Distributions* **22**, 1276-1287.

Chapuis MP, Estoup A (2007) Microsatellite null alleles and estimation of population differentiation. *Molecular Biology & Evolution* **24**, 621-631.

Cornuet JM, Ravigné V, Estoup A (2010) Inference on population history and model checking using DNA sequence and microsatellite data with the software DIYABC (v1.0). In: *BMC Bioinformatics*, p. 11.

Cornuet JM, Santos F, Beaumont MA, Robert C, Marin J-M, Balding DJ, Guillemaud T, Estoup A (2008) Inferring population history with DIY ABC: a user-friendly approach to Approximate Bayesian Computation. *Bioinformatics* **24**, 2713-2829.

Estoup A, Lombaert E, Marin JM, Guillemaud T, Pudlo P, Robert CP, Cornuet JM (2012) Estimation of demo-genetic model probabilities with Approximate Bayesian Computation using linear discriminant analysis on summary statistics. *Molecular Ecology Resources* **12**, 846-855.

Fang CG (2016) The occurrence regularity and pollution-free control technology of peach fruit moth *Caposina sasakii* (Lepidoptera: Carposinidae) in Xinbin mountains. *Protection Forest Science and Technology*, 109-110.

**Abstract:** *Carposina sasakii* is an important pest of pear fruit in Xinbin mountain area (in Liaoning province, northeastern China), the rate of bored fruits is up to 60%-90% under improper control, which causes serious economic losses. Peak of unearthed period of *Carposina sasakii* is in mid to end of June. Both egg-laying phase and peak of hatching are in first half of July. In the early period of unearthed or when rate of bored fruits is up to 1%, spraying pesticide with low toxicity on area and tree should be combined.

Hua L, Hua BZ (1995) Preliminary study on the host-biotypes of peach fruit borer. *Acta Phytophylacica Sinica* **22**, 165-170.

**Abstract:** Every one of the different host-biotypes of peach fruit borer (PFB) *Carposina niponensis* Walsingham is adaptable to the phenophases, fruit shapes and textures of each one’s own host, thus the behavior characteristics are obviously different from each other. Under suitable room temperature and humidity conditions, the beginning and peak times of that the overwintering PFB larvae got from jujube and wild tree come out from the ground are 8 and 20 days respectively later than that from apple tree, and the field occurrence time of adults of the former two biotypes are also 20 days later. Under constant temperature condition, the duration of prepupation and pupation stages of both the former two biotypes are shorter than the latter. All the positions of oviposition of adults, and boring in and out of larvae and their living spans in fruit among the three host-biotypes are different. The size of PFB moth from wild jujube is smaller, but the male genitals of the three biotypes are similar. All the hybrid females can lay eggs and the eggs can hatch. The esterase isoenzyme spectra of diapausing larvae and overwintering larvae just come out from the ground of three host-biotypes are significantly different from each other.

Hua L, Hua BZ, huang WL (1998) The bionomics of peach fruit borer damaged on apricot tree. *Acta Phytophylacica Sinica* **25**, 141-144.

**Abstract:** The peach fruit borer (PFB), *Carposina sasakii* Matsumura is mostly one generation per year on apricot tree in Shaanxi Province. The time when its overwintering larvae come out of the ground and occurrence period of adults are all half a month or more earlier than those of the same insect pest damaged on apple tree. By the test results in 1993-1996, the diapause rate of first generation larvae was nearly up to 99% or so, i.e. closed to obligatory diapause. Under 21°C indoor temperature and 14.5-15.5 h/day of photo times, the diapause of FFB larvae was nearly not effected by the photoperiods. The esterase isozymes of its overwintering larvae and the larvae just came out of the ground were significantly different from those of PFB on apple. Some suggestions on the control of PFB on apricot were proposed.

Lombaert E, Guillemaud T, Lundgren J, Koch R, Facon B, Grez A, Loomans A, Malausa T, Nedved O, Rhule E, Staverlokk A, Steenberg T, Estoup A (2014) Complementarity of statistical treatments to reconstruct worldwide routes of invasion: the case of the Asian ladybird *Harmonia axyridis*. *Molecular Ecology* **23**, 5979-5997.

Ryman N, Palm S (2006) POWSIM: a computer program for assessing statistical power when testing for genetic differentiation. *Molecular Ecology Notes* **6**, 600-602.

Wang YZ, Cao LJ, Zhu JY, Wei SJ (2016) Development and characterization of novel microsatellite markers for the peach fruit moth *Carposina sasakii* (Lepidoptera: Carposinidae) using next-generation sequencing. *International Journal of Molecular Sciences* **17**, 362.
